# Hypoxia Enhances Fusion of Oral Squamous Carcinoma Cells and Epithelial Cells Partly via the Epithelial–Mesenchymal Transition of Epithelial Cells

**DOI:** 10.1155/2018/5015203

**Published:** 2018-02-08

**Authors:** Chun-ming Huang, Ting-lin Yan, Zhi Xu, Meng Wang, Xiao-cheng Zhou, Er-hui Jiang, Ke Liu, Zhe Shao, Zheng-Jun Shang

**Affiliations:** ^1^The State Key Laboratory Breeding Base of Basic Science of Stomatology (Hubei-MOST) & Key Laboratory for Oral Biomedicine Ministry of Education, Wuhan University, Wuhan, China; ^2^Department of Stomatology, Union Hospital, Tongji Medical College, Huazhong University of Science and Technology, Wuhan, China; ^3^Department of Oral and Maxillofacial-Head and Neck Oncology, School and Hospital of Stomatology, Wuhan University, Wuhan, China

## Abstract

Increasing evidence and indications showed that cell fusion is crucial in tumor development and metastasis, and hypoxia, a closely linked factor to tumor microenvironment, which can lead to EMT, induces angiogenesis and metastasis in tumor growth. However, the relationship between hypoxia and fusion has not been reported yet. EMT will change some proteins in the epithelial cell surface and the changes of proteins in cell surface may increase cell fusion. This study found that hypoxia promotes the spontaneous cell fusion between Oral Squamous Carcinoma Cells (OSCCs) and Human Immortalized Oral Epithelial Cells (HIOECs). At the same time, Hypoxia can lead to EMT, and hypoxia-pretreated HIOECs increased fusion rate with OSCC, while the fusion rate was significantly reduced by DAPT, a kind of EMT blocker. Therefore, epithelial cells can increase spontaneously cell fusion with OSCC by EMT. Our study may provide a new insight to link among tumor microenvironment, cell fusion, and cancer.

## 1. Introduction

Cell fusion, also known as cell hybrid, is a highly accurate and well-organized process in development and homeostasis [1–3]. This process is a specialized form of membrane fusion and a state of nuclear fusion and DNA communication [4]. Although cell fusion was proposed 100 years ago [5], the topic received minimal attention. Cell fusion has been recently broached as an important power in tumor progress and metastasis [5, 6] and occurs between somatic cells, tumor cells, and somatic cells × tumor cells [7–14]. It is an important part of normal development and a considerable factor in pathological process. However, the mechanisms underlying cell fusion and its link to tumor metastasis remain poorly explored.

Tumor growth in tumor microenvironment is influenced by many factors, such as hypoxia, inflammation, and immune response [11, 15–18]. Hypoxia is an essential condition of tumor microenvironment that is associated with tumor metastasis and poor prognosis [15, 19]. To date, many studies have reported the mechanisms and signaling pathways underlying hypoxia and tumor metastasis, including HIF-*α* [20], NOTCH/SOX2 [21], and PI3K/Akt [22]. Many researchers reported that hypoxia promotes cell–cell adhesion and interaction between tumor and somatic cells [2]. Hypoxia also upregulates the expression of adhesive proteins, such as integrin [2], intercellular adhesion molecule 1 [23], and fibronectin [24, 25]. Nevertheless, cell–cell interaction and adhesion are the key processes prior to cell fusion. Simultaneously, cell fusion promotes tumor progression and metastasis [5, 14]. Hence, we speculated that some links are present among hypoxia, cell fusion, and tumor progression. We also hypothesized that hypoxia enhances cell–cell fusion and further accelerates the progress and metastasis of tumor.

Epithelial–mesenchymal transition (EMT) is a morphogenetic change where epithelial cells lose their polarity and are converted into mesenchymal phenotypes [26]. EMT is a vital event during wound healing, embryonic development, and tumor metastasis [27–29]. Recently published studies have shown that EMT is closely associated with tumor microenvironment [30], inflammation [31], tumor progression and metastasis, and cell–cell interaction [27, 29]. As an important factor affecting tumor microenvironment, hypoxia powerfully promotes EMT [3, 22, 26, 32–34]. Kaneko et al. [22] reported that hypoxia regulates and promotes EMT in oral squamous cell carcinoma via the PI3K/Akt signaling pathway. Reports also revealed that colon cancer [33], ovarian cancer [21], and laryngeal cancer [3] are regulated by hypoxia via diverse signaling pathways. However, the relationship among hypoxia, EMT, and cell fusion remains unknown. Hypoxia can tie cell fusion and EMT together. Proteins or molecule in cell surface would change, when EMT happened. Although the rate of spontaneous cell fusion was relatively low, cell fusion had a great effect on tumor metastasis and invasion, so the changes of proteins or molecules in cell surface are very important. Thus, the study of what cell fusion rate can increase by hypoxia via EMT was meaningful to help to study tumor metastasis and invasion.

Thus, we aimed to uncover the relationship of cell fusion to hypoxia and EMT. We cocultured CAL-27 with HIOECs and found that spontaneous cell fusion occurs between OSCC cells and HIOECs. The CAL-27 and HIOEC cocultured system was treated with hypoxia, and the fused cells were analyzed. Results showed that the fusion rate increased compared with the untreated group. In addition, the indicators of EMT changed in HIOECs. The hypoxia group fusion rate increased. When EMT was partially blocked by DAPT, the fusion rate decreased significantly. In short, we initially proved that hypoxia enhances the spontaneous cell fusion between OSCC cells and HIOECs partially via inducing the EMT of HIOECs.

## 2. Materials and Methods

### 2.1. Cell Lines and Cell Culture

The human OSCC lines, CAL-27, were kindly donated by Professor Zhuan-Bian, which were purchased from American Type Culture Collection (ATCC, Manassas, VA, US). The OSCC was cultured in Dulbecco's modified Eagle's medium (DMEM) high glucose (Hyclone, UT, USA) and added with 10% FBS (Gibco, Carlsbad, Calif, USA). Human Immortalized Oral Epithelial Cells (HIOECs) were kindly provided by Professor Cheng-zhang Li and Doctor Zhen-Zhang. The HIOECs were cultured in KGM gold (Lonza, Walkersville, MD) which was added with 5% fetal bovine serum (FBS) and KGM gold growth factor mixture. And all of the control group cells were cultured in an incubator which was kept at 37°C and contained 5% CO_2_. The hypoxia atmosphere was kept at 37°C in an incubator with 1.0% O_2_. HIOECs were placed under hypoxia (1% O_2_) or normoxia (5% CO_2_) for 24 h; EMT was blocked by 5 *μ*M DAPT (Catalog number T6202, TargetMol, China).

### 2.2. Cell Transduction, Screening Stable Cells, and Coculture

The Recombination lentiviral vectors of GFP- and RFP- were purchased from GenePharma, Shanghai, China. Then the cultured CAL-27 and HIOEC were added with lentiviral supernatants which contained GFP and RFP vector. After transduction for 72 h, the RFP-/GFP-CAL-27 and GFP-/RFP-HIOEC were selected with 10 *μ*g/ml puromycin. The efficiency of transduction was screened by immunofluorescent (CarlZeiss, Germany) and FACS (CyteFLEX, Beckman Coulter, USA).

### 2.3. Confocal Observation

RFP-CAL-27 and GFP-HOIEC were cocultured for 3 days and washed three times with PBS for 5 minutes each and then fixed with 4% paraformaldehyde for 15 min at room temperature, stained with DAPI at room temperature for 5 min, and washed three times with PBS for 5 minutes each. And confocal microscopy (Olympus FV1200, Japan) observed the fused cells.

### 2.4. Protein Extraction and Western Blot Analysis

Total protein of HIOECs was extracted by using M-PER (Pierce Inc, USA) supplemented with Protease inhibitor and phosphatase inhibitor on ice. The quantity of every sample was tested by BCA protein assay kit (Thermo Fisher Scientific Inc. USA). Then mixed liquor was formed by accessed loading buffer (5x) and heated for 10 minutes at 95°C. In the following, aliquots of 10 *μ*g of protein were added to 8% sodium dodecyl sulfate-polyacrylamide gel electrophoresis for 30 mins at 60 V and 1 h at 110 V. Afterwards, proteins were transferred to polyvinylidene difluoride (PVDF) membrane in transfer buffer for 2 h at 200 mA. The membranes were blocked with 5% nonfat milk in Tris-buffered saline which contained 0.05% Tween 20 at room temperature for 2 h. Then the membranes were incubated with anti-GAPDH antibody (1 : 6000) (Proteintech, Wuhan, China), anti-Slug antibody (1 : 1000, CST), anti-Vimentin antibody (1 : 1000, CST), anti-E-cadherin antibody (1 : 1000, CST), and anti-N-cadherin antibody (1 : 1000, CST) overnight at 4°C. Then the bound antibodies were tested by horseradish peroxidase-conjugated, anti-rabbit IgG or anti-mouse IgG (Pierce Chemical, Rockford, IL, USA). To confirm the consequences, the analysis of Western blot was repeated at least three times.

### 2.5. FACS Assay

RFP-CAL-27 and GFP-HIOECs were cocultured with the mix of 1 : 1 for 3 groups. Control group: all cells were cultured in an incubator which was kept at 37°C and contained 5% CO_2_. The hypoxia group: the hypoxia atmosphere was kept at 37°C in an incubator with 1% O2. Hypoxia-pretreated group: HIOECs were pretreated with hypoxia (37°C, 1% O2) for 24 h with or without DAPT (5 *μ*M); then these HIOECs were cocultured with CAL-27 in an incubator which was kept at 37°C and contained 5% CO2. Then results were gathered for days 1 and 3. The double-labeled fluorescent staining positive cells were observed in the fluorescence microscope (Leica, Wetzlar, Germany).

### 2.6. Statistical Analysis

All of the statistical analyses were managed by Student's *t*-test, one-way ANOVA, GraphPad Prism 5.01 (GraphPad Software, USA), and FlowJo 7.6.1 (FlowJo Software, USA). Statistical data were expressed as mean ± standard deviation. The criterion of statistical significance was that *p* values < 0.05. All results were repeated at least three times.

## 3. Results

### 3.1. Identification of Fusion Cell

HIOECs were transfected using fluorescence recombinant lentiviral vectors with RFP- and GFP- to determine the fusion events between CAL-27 and HIOECs by intuitive observation. RFP-CAL-27 and GFP-HIOECs were developed, and cells with fluorescent staining were observed under a fluorescence microscope and then screened with puromycin. After culturing for 72 h, the stable transfected cells were sorted by FACS. The stable transfected RFP-CAL-27 and GFP-HIOECs were cocultured with a ratio of 1 : 1. After steady culture for 1 and 3 days, the double-labeled fluorescent staining cells were observed and counted by three people from different random fields. And the confocal microscope was used to make sure of the fusion between the two kinds of cells.

**Figure 1:**
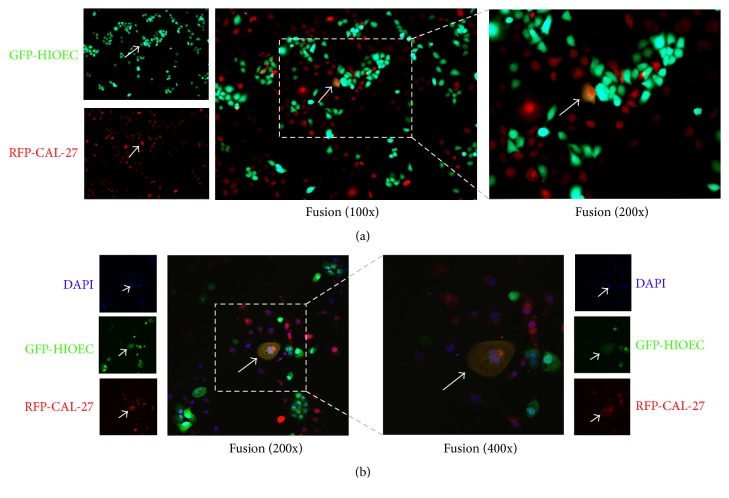
(a) Representative images of fused cells between RFP-CAL-27 and GFP-HIOECs (100x and 200x). (b) Representative confocal images of fused cells between RFP-CAL-27 and GFP-HIOEC (200x and 400x). (Green fluorescence protein: HIOEC; Red fluorescence protein: CAL-27).

As shown in Figure 1, cells with double fluorescent staining exhibited orange-like color in the coculture system (Figure 1(a)). And the fused cell, between RFP-CAL-27 and GFP-HIOECs, can be seen by both the red fluorescence and green fluorescence separately. In order to observe cell fusion accurately, confocal microscopy was used to observe fused cells. The double-labeled fluorescent staining cells were observed (Figure 1(b)).

### 3.2. Cell Fusion between CAL-27 and HIOECs Was Enhanced under Hypoxic Conditions

Hypoxia is an important factor affecting tumor microenvironment. Some studies revealed that hypoxia accelerates the development and progression of tumors. The present study is a preliminary attempt to elucidate the relationship between hypoxia and cell fusion. Stable transfected RFP-CAL-27 was cocultured with GFP-HIOECs, and the cocultured system was exposed to hypoxia with 1.0% O_2_, 37°C. The control group was cultured in an incubator at 5% CO_2_, 37°C.

**Figure 2:**
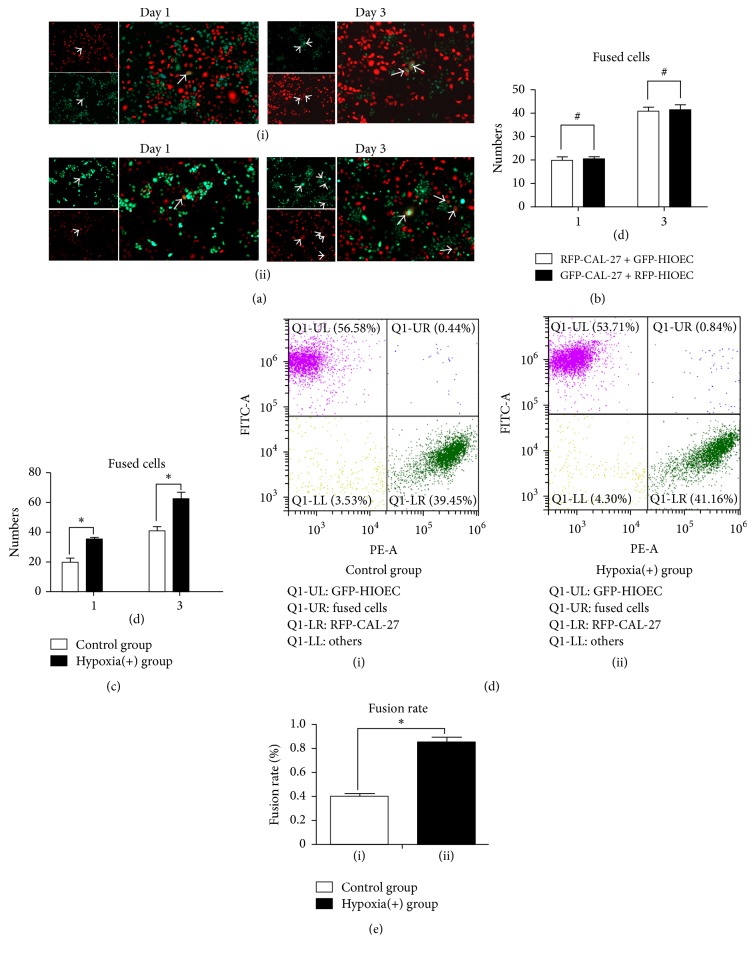
(a)(i)-(ii) Representative images of cell fusion (RFP-CAL-27 × GFP-HIOEC) between control group and hypoxia (+) group of days 1 and 3. (b) Number of fused cells of RFP-CAL-27 × GFP-HIOECs and GFP-CAL-27 × RFP-HIOECs cocultured system by artificial counting method. ^#^*p* > 0.05. (c) Number of fused cells in RFP-CAL-27 × GFP-HIOECs cocultured system by artificial counting method. ^*∗*^*p* < 0.05. (d) Fusion rates of RFP-CAL-27 × GFP-HIOECs by FACS. (e) Statistical analysis of fusion rate of RFP-CAL-27 × GFP-HIOECs. ^*∗*^*p* < 0.05.

As shown in Figure 2, the number of fused cells was measured using the artificial counting method conducted by three different people in three random fields. The fusion rate was measured by FACS.  Figure 2(a) showed the representative images of cell fusion between the control group (Figure 2(a)(i)) and the hypoxia-stimulated group (Figure 2(a)(ii)). When the cocultured system was cultured for 1 and 3 days, the number of orange-like colored fused cells in the hypoxia group significantly increased compared with that in the control group.  Figure 2(b) revealed no difference in the fusion rate between the cocultured systems of RFP- CAL-27 × GFP-HIOECs and GFP-CAL-27 × RFP-HIOECs. This result demonstrated that the fluorescence recombinant lentiviral vectors with RFP- and GFP- did not affect the fusion rate of the cocultured system.  Figure 2(c) presents the number of fused cells determined by artificial cell counting. The number of fused cells between CAL-27 and HIOECs in the hypoxia (+) group increased by up to nearly 1.5-folds compared with control group, and the results showed significantly statistical differences. The fusion rates of the hypoxia (+) and control groups were determined by FACS in Figures 2(d) and 2(e). Hypoxia increased the percentage of bifluorescent cells up to 0.85% ± 0.06% compared with the control group (0.39% ± 0.08%), and the results showed significantly statistical differences.

### 3.3. Fusion Could Be Enhanced between CAL-27 and Hypoxia-Pretreated HIOECs Partly by EMT

EMT and hypoxia are closely associated with tumor progression and metastasis. However, only few reports revealed the relationship among hypoxia, EMT, cell fusion, and tumor metastasis. We hypothesized that hypoxia partly enhances fusion between CAL-27 and HIOECs through EMT. HIOECs were cultured in an incubator with 1.0% O_2_ for 24 h, and EMT-associated indexes, including E-cad, N-cad, Vimentin, and Slug, were measured using Western blot. Hypoxia-pretreated HIOECs were cultured with CAL-27 in an incubator with 5.0% CO_2_. After being cocultured for 1 and 3 days, fused cells were calculated using the artificial counting method.

**Figure 3:**
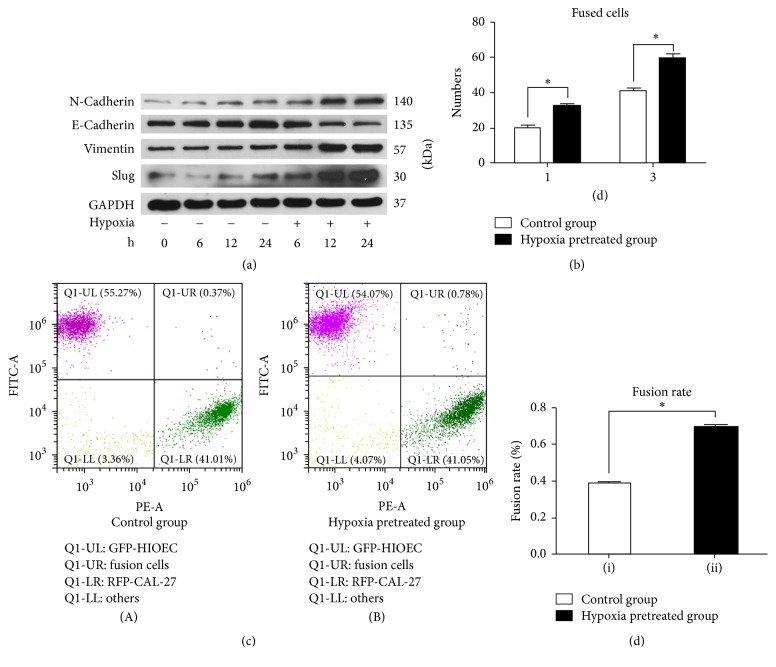
(a) The expression of EMT-associated indexes including N-cadherin, E-cadherin, Vimentin, and Slug with/without hypoxia stimulation. (b) The number of fused cells by cell counting with/without the pretreated of hypoxia after 1 and 3 days. (^*∗*^*p* < 0.05). (c) The fusion rate of the hypoxia pretreated group and the control group by FACS. (d) Statistical analysis of fusion rate of the hypoxia pretreated group and the control group (^*∗*^*p* < 0.05).

As shown in Figure 3(a), HIOECs were pretreated in 1% O_2_ atmosphere for 6, 12, and 24 h. Western blot was conducted to detect the content of EMT-associated indexes, including N-cadherin, E-cadherin, Vimentin, and Slug. The expression level of Slug increased compared with the control group (normoxia group), whereas the expression levels of Vimentin and N-cadherin remarkably increased compared with the normoxia group. By contrast, the expression level of E-cadherin evidently reduced compared with the control group. Basing from these results, we concluded that HIOECs were converted into mesenchymal cells under the stimulation of hypoxia.  Figure 3(b) shows the results of cell fusion by cell counting with/without the pretreated of hypoxia after 1 and 3 days. GFP-HIOECs were pretreated in 1% O_2_ atmosphere and then cocultured with RFP- CAL-27 at a ratio of 1 : 1 in normal oxygen environment for 1 and 3 days. The same amount of 1 : 1 RFP-CAL-27 and GFP-HIOECs was cocultured in an incubator with normal oxygen environment to serve as the control. The number of fused cells evidently increased compared with the control group (1.55 ± 0.10 fold). This result indicates that the HIOECs pretreated with hypoxia showed better fusion with CAL-27 compared with the normal HIOECs without any irritation. Figures 3(c) and 3(d) show the fusion rates of the hypoxia-pretreated group (0.39% ± 0.03%) and the control group (0.78% ± 0.02%) by FACS. The fusion rate of the hypoxia-pretreated group was significantly improved compared to that of the control group (*p* < 0.05).

### 3.4. Cell Fusion Was Decreased between OSCC Cells and HIOECs by DAPT via Blocking EMT of HIOECs

**Figure 4:**
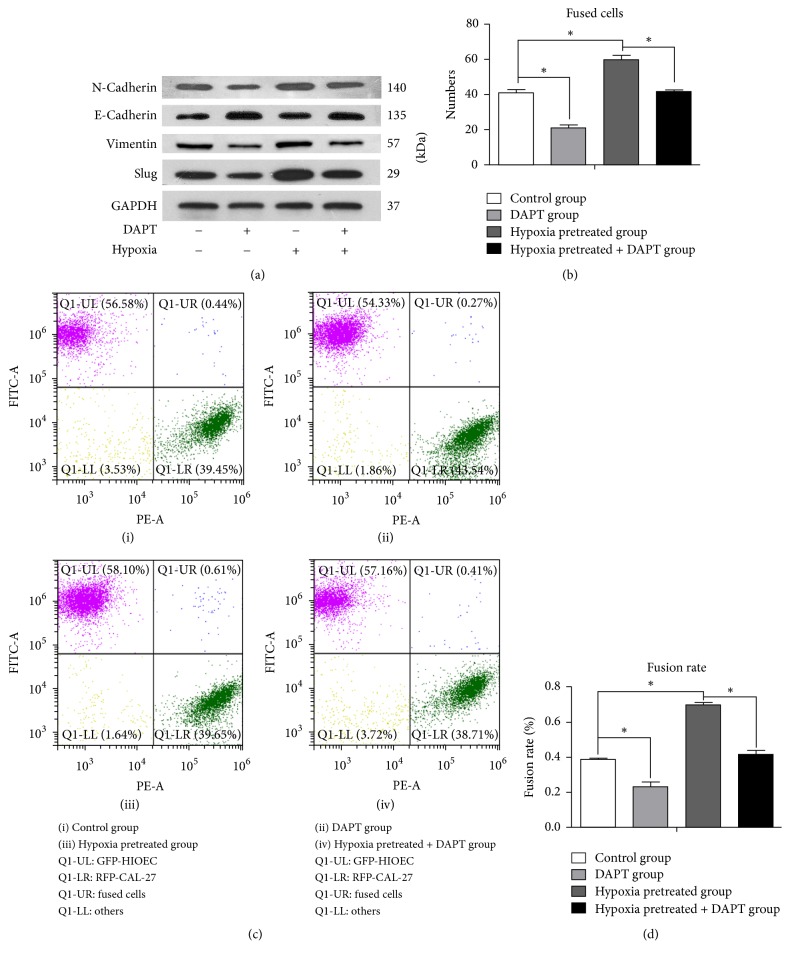
(a) The expression of EMT-associated indexes including N-cadherin, E-cadherin, Vimentin, and Slug with/without hypoxia or DAPT stimulation. (b) The number of fused cells by artificial cell counting with/without the pretreated of hypoxia or DAPT for 3 days. (c) The fusion rate of the pretreated group and the control group by FACS. (i) Hypoxia (−), DAPT (−); (ii) hypoxia (−), DAPT (+); (iii) hypoxia (+), DAPT (−); (iv) hypoxia (+), DAPT (+). (d) Statistical analysis of fusion rate of the pretreated group and the control group (^*∗*^*p* < 0.05).

EMT-associated indexes, including E-cad, N-cad, Vimentin, and Slug, were measured using Western blot. It was indicated that hypoxia induce the EMT of HIOECs (Figure 4(a)). Then HIOECs were treated with DAPT ((N-[N-(3,5-difluorophenacetyl)-l-alanyl]-S-phenylglycine t-butyl ester)) (5 *μ*M), an EMT inhibitor, under hypoxia environment (1.0% O_2_) for 24 h. As shown in Figure 4(a), Western blot demonstrated that the E-cadherin expression was decreased, whereas the expression levels of Slug, Vimentin, and N-cadherin remarkably increased compared with the DAPT-unpretreated group. Thus, it was concluded that EMT could be partly blocked by DAPT.

After that, GFP-HIOECs were cocultured with RFP-CAL-27 cells for 3 days. Fused cells were calculated and analyzed using the artificial counting and FACS method. By artificial cell counting, it was indicated that fused cell numbers were decreased in DAPT-pretreated group compared with DAPT-unpretreated group under normoxia environment. Furthermore, DAPT could reverse the increased cell fusion ability under hypoxia treatment (Figure 4(b)). It was also showed that the cell fusion rate was reduced in DAPT-pretreated group compared with negative control. Meanwhile, DAPT could also significantly counteract the cell fusion rate increasing in hypoxia treatment group. (Figures 4(c) and 4(d)).

## 4. Discussion

Cell fusion, also called cell hybrid, was proposed 100 years ago [1]. Although this topic was introduced for many years, this phenomenon is still poorly understood. Researchers have recently found that cell fusion is closely associated with tumor metastasis [14]. In our previous study, we found that spontaneous cell fusion between SCC-9 and HUVECs partly occurs via the Wnt/*β*-catenin signal pathway. Fusion is enhanced by inflammatory factors such as TNF-*α* [9, 11]. Other studies showed that tumor microenvironment is closely related to tumor metastasis. Studies on inflammatory factors [18, 35], microvesicles [30], and hypoxia [3, 19] now focus on cell fusion and tumor metastasis. In the present study, we further discuss the relationship between hypoxia and cell fusion. We found that hypoxia, an important factor influencing tumor microenvironment, enhances the fusion between OSCC and pretreated HIOECs.

Infinite proliferation, also called immortalization, is an important hallmark of cancer [17]. Tumor cells achieve metastasis via evading growth suppressors, resisting cell death, inducing angiogenesis, enabling replicative immortality, and sustaining proliferative signaling [17]. Aneuploidy [36], heterogeneity [37], and angiogenesis [38] are also important for tumor cells. Previous studies reported that the formation of aneuploidy and heterogeneity results from errors in cell division and differentiation [36, 37]. However, recent studies have presented other reasons. Cell fusion is one of the elegant theories [6, 14, 39]. Hybrid cells acquire the genetic materials of both parental cells through the fusion of the membrane and the nucleus [4, 14, 40–42]. We have verified that spontaneous cell fusion between tumor and endothelial cells is enhanced by inflammatory factors such as TNF-*α*. HIOECs feature stronger viability and unlimited growth compared with HUVECs [43, 44]. Therefore considering the spontaneous fusion of OSCC and HIOECs, the daughter hybrid cells may have more potential for metastatic and survival ability than parents

Tumor microenvironment is important in tumor progression and metastasis. Hypoxia is related to tumor metastasis and EMT [3, 19, 22, 26, 32, 33]. We pretreated HIOECs with hypoxia for 24 h and detected the expression of EMT correlative indexes. The expression levels of N-cadherin, Vimentin, and Slug were evidently increased, whereas that of E-cadherin was significantly reduced. We cocultured these hypoxia-pretreated HIOECs with CAL-27 for 1 and 3 days. We found that the fusion event increased by about almost 1.5-folds compared with the control group. DAPT could block the EMT in gastric cancer [45] and epithelial ovarian cancer [46]. And EMT of HIOECs could be blocked by DAPT in our study. And when EMT was blocked by DAPT, the fusion event significantly reduced. This finding suggests that hypoxia partly enhanced the fusion between HIOECs and CAL-27 via EMT. The state of mesenchyme strengthens cell fusion compared with the epithelial condition.

Hypoxia contributes to the development of EMT and changes in the proteins of the cell surface, thus adapting to changes in the microenvironment. EMT reduces the expression of E-cad and reduces the connection between epithelial cells and epithelial cells, which makes it easier for epithelial cells to separate from surrounding tissue cells, and it provides the conditions for the invasion and metastasis of cancer. This paper demonstrated that the fusion rate could significantly improve when an EMT occurred in HIOEC. And when EMT was blocked or reversed by DAPT, the expression of E-cad increased, and the ability of cell adhesion was enhanced. It would be more difficult for epithelial cells to separate from surrounding tissue cells, and the fusion rate of occurrence was also reduced. The EMT could promote the invasion and metastasis of tumor, as cell fusion did. The fusion cells coexpress differential phenotypes of both parents [47], just as a tumor cell has an EMT-like change by cell fusion. EMT promoted cell fusion, to a certain extent, which suggested that tumor microenvironment changes tumor cells into a more adaptive way for tumor cell survival, providing a boost for tumor invasion and metastasis. Therefore, blocking tumor EMT during tumor treatment may be helpful in inhibiting tumor invasion or metastasis. The signaling pathway where DAPT decreases cell fusion by blocking EMT is unclear now, so the much further research on the signaling pathway needs to be done in the future. At the same time, the mechanism of cell fusion to promote tumor invasion and metastasis is worthy of further study because of the low rate of spontaneous fusion.

In conclusion, we found that OSCCs and HIOECs can spontaneously fuse. And hypoxia enhances the spontaneous cell fusion between OSCCs and HIOECs partially via inducing the EMT of HIOECs.
